# A Matter of Font: Visual Word Form Area Responses to Brand Names Are More Sensitive to Font Than to Letter Case Changes

**DOI:** 10.1162/NOL.a.268

**Published:** 2026-07-01

**Authors:** Melanie Labusch, Manuel Perea, Tatiana Davydova, María Baena Pérez, Cristina Cano Melle, Esteban Villar Rodriguez, Eric Rivero Zaragoza, Victor Costumero

**Affiliations:** Department of Methodology and ERI-Lectura, Universitat de València, Valencia, Spain; Health Research Institute La Fe, Valencia, Spain; Centro de Investigación Nebrija en Cognición (CINC), Nebrija University, Madrid, Spain; Department of Basic and Clinical Psychology and Psychobiology, Universitat Jaume I, Castellón de la Plana, Spain

**Keywords:** brand names, fMRI, neuroimaging, reading, visual word form area, visual word recognition

## Abstract

Lexical access to common words is remarkably robust to changes in surface format (e.g., font, case, color, size), supporting abstractionist models in which orthographic codes are largely invariant to perceptual details. However, recent behavioral experiments have shown that brand names, a type of lexical item that is typically encountered in a highly consistent visual format, are sensitive to modifications of surface format (Labusch, Duñabeitia, & Perea, 2024; Perea et al., 2022). This research has largely focused on behavioral measures, leaving open questions about the neural mechanisms underlying brand name recognition. Here, we used fMRI to examine whether the visual word form area (VWFA) and related regions are sensitive to font and letter case modifications in brand names in a semantic categorization task (brand related to transportation or not). Participants viewed brand names presented either in their typical visual format, or with a modified font or case. Font modifications slowed responses and elicited increased VWFA activation relative to intact logos, whereas case modifications also slowed responses but did not reliably modulate activity in the canonical VWFA ROI. This dissociation constrains strong claims of strict format invariance in the VWFA, while remaining compatible with hierarchical or interactive accounts in which some ventral occipitotemporal populations retain sensitivity to learned surface forms. Rather than supporting a categorical claim of case invariance, the present data indicate stronger sensitivity to font than to letter case when a perceptual format is consistently tied to lexical identity.

## INTRODUCTION

Reading written words in alphabetic scripts initiates a cascade of neural computations, beginning with low-level visual processing in the occipital cortex and advancing progressively to higher-order lexical and semantic representations along the ventral occipitotemporal pathway (Dehaene et al., 2005; Jobard et al., 2003; Saygin et al., 2016; Vinckier et al., 2007). Within this pathway, the visual word form area (VWFA) in the left fusiform gyrus (Cohen et al., 2000, 2002) provides rapid access to orthographic codes.

Although the VWFA has been widely studied, the precise representational format of the orthographic codes it supports remains under debate (see Dȩbska et al., 2023; Dehaene et al., 2005; Li et al., 2024; Price & Devlin, 2011; Zhou et al., 2019). One dominant view assumes abstract letter identities that are invariant to surface variation and derived through a process of normalization (Bowers, 2000; Dehaene et al., 2005; Grainger et al., 2008). An alternative view is provided by instance-based accounts, who propose memory traces that retain specific perceptual features encoded during learning (Goldinger, 1998; Jamieson et al., 2022; Tenpenny, 1995). Critically, these two broad classes of models make different predictions about how much influence surface regularities should retain once orthographic identity is accessed. For items that are consistently experienced in the same visual format, such as brand names, strict versions of abstractionist accounts predict that responses at more abstract levels of orthographic coding should show limited dependence on those surface regularities, whereas instance-based accounts more naturally allow learned perceptual regularities to remain functionally relevant. We address this issue using familiar brand names, while recognizing that responses in a canonical VWFA ROI need not map onto a single representational level. Accordingly, we tested whether responses in this ROI remain stable across changes in the visual format of brand names or instead show sensitivity to learned aspects of their customary logotypes.

Most biologically inspired models of visual word recognition assume that visual information (e.g., font, size, color, and case of letters) is normalized throughout processing, such that word recognition relies on abstract letter identities that are invariant to perceptual features (*abstractionist models*, see Dehaene et al., 2005; Grainger et al., 2008). This normalization enables efficient mapping of variable inputs to lexical entries. Empirical support comes from functional neuroimaging studies investigating repetition suppression effects in the VWFA: in a masked priming letter detection experiment (“does the word include the letters A or E?”), target words like *edge* resulted in comparable reductions in VWFA activation whether briefly preceded by same-case (edge-edge) or different-case (EDGE-edge) primes (Dehaene et al., 2004, see also Polk & Farah, 2002). Likewise, Zhou et al. (2019) reported partially separable font-invariant and font-sensitive neural populations in the occipitotemporal cortex during repeated-word processing (see Gauthier et al., 2000, for similar findings with individual letters). Accordingly, the available evidence does not support a simple all-or-none view of format invariance; rather, it suggests that surface-form sensitivity can coexist with more abstract coding within the ventral occipitotemporal cortex. Earlier work has therefore often been interpreted as indicating that VWFA responses are relatively robust to visual format changes, such as font or case (see Jacobs et al., 1995, for behavioral evidence; see also Chauncey et al., 2008; Vergara-Martínez et al., 2015, 2020, for electrophysiological evidence), without necessarily implying complete insensitivity to perceptual format. At the same time, the VWFA is modulated by lexical variables such as frequency (Kronbichler et al., 2004), suggesting that its representations are not purely visual but engage with higher-level information. In interactive accounts of the ventral occipitotemporal cortex, sensitivity to visual format can be understood in terms of a prediction error: neural responses should increase when the incoming visual input departs from the expected visual form of a familiar item (see Price & Devlin, 2011).

Recent neuroimaging evidence from German, a language in which all common nouns are capitalized (e.g., *Ball* [ball] vs. *ball*), challenges the view that VWFA processing relies solely on abstract letter identities. In an event-related fMRI lexical decision experiment, Wimmer et al. (2016) presented German nouns like *Ball* and adjectives like *blau* in their familiar letter case format (i.e., nouns with initial capitalizations and adjectives in lowercase) or in an unfamiliar case format (*ball* and *Blau*). They found greater VWFA activation for words presented in an atypical case format than in their canonical case. These results suggest, consistent with previous behavioral evidence (Jacobs et al., 2008; Labusch et al., 2022; see also Peressotti et al., 2003, for evidence with proper nouns), that VWFA activation is modulated by deviations from canonical visual configurations. Thus, these findings highlight that the visual familiarity of a written word, not only their abstract identity, can shape neural responses in the VWFA.

Brand names (i.e., lexical items typically encountered in highly consistent visual formats) offer a powerful test case for probing the role of surface features in the VWFA. Unlike common words, where abstraction is arguably driven by the need to map highly variable perceptual formats (e.g., house, HOUSE, *house*) onto a single lexical entry, brand names usually appear in a fixed letter case (e.g., *IKEA* in uppercase, *adidas* in lowercase) and are tied to a distinctive typeface (e.g., the Coca-Cola font). Consequently, there may be less need to abstract away from those surface features. This visual consistency makes brand names a useful test case for asking whether orthographic representations can remain sensitive to features such as font when those features are consistently associated with identity. Prior research suggests that such consistency may lead to mental representations that incorporate surface-level features (Labusch, Duñabeitia, & Perea, 2024; Labusch, Perea, et al., 2024; Pathak et al., 2019; Perea et al., 2021, 2022). Specifically, recent behavioral and ERP experiments show slower responses and distinct electrophysiological patterns for brand names presented in altered font or case formats relative to the canonical format (Gontijo et al., 2002; Gontijo & Zhang, 2007; Labusch, Duñabeitia, & Perea, 2024; Perea et al., 2015, 2022; see also Labusch & Perea, 2025, for eye tracking evidence). These neural and behavioral differences extend to late ERP components (approximately 650 ms), typically associated with lexical-semantic integration (Labusch et al., 2026).

Taken together, these findings can be interpreted within instance-based models (e.g., Goldinger, 1998; Tenpenny, 1995), in which episodic traces may incorporate perceptual details, but they are not exclusive to such accounts. Specifically, brand names may be recognized through the retrieval of memory traces that include both letter identities and visual forms. For common words, which appear in diverse visual formats, episodic traces converge on increasingly abstract representations over time (Bowers, 2000). However, brand names may reduce the pressure toward abstraction-by-averaging. Their consistency across time and media can allow perceptual features such as font and case to remain traceable and functionally relevant within their neural representations (Labusch, Duñabeitia, & Perea, 2024; Labusch & Perea, 2025). This idea fits well with the interactive accounts of the ventral occipitotemporal cortex (Price & Devlin, 2011), in which word recognition involves the integration of bottom-up input with top-down expectations. In this framework, brand names presented in their canonical visual formats should prompt strong top-down predictions, whereas deviations from those expected formats should generate greater prediction error and consequently increased neural activity. Critically, no fMRI study has directly tested whether surface features alter VWFA activation for brand names, leaving open whether this region’s presumed invariance breaks down when familiar formats are disrupted.

The present experiment addresses this gap by examining whether changes in font or case increase VWFA activation in comparison to the canonical format of brand names. Notably, in the Wimmer et al. (2016) fMRI study in German (comparing *ball* vs. *Ball* [ball, in English] and *Blau* vs. *blau* [blue, in English]), case modifications were not purely visual. Capitalization also signaled grammatical class (e.g., nouns vs. adjectives), potentially engaging morphosyntactic processes and orthographic conventions in addition to visual-orthographic processing. In contrast, we manipulate font and case while holding both the lexical identity and semantic role of the brand names constant, providing a cleaner test of perceptual sensitivity in the VWFA. Furthermore, Wimmer et al. used a lexical decision task, which can be strongly influenced by visual and orthographic familiarity (see Perea et al., 2020). To reduce the contribution of such low-level familiarity and ensure that participants access word meaning, the present experiment employs a semantic categorization task (“Does the brand name correspond to a means of transportation?”).

To address this question, we employed an event-related semantic categorization task (see Labusch, Perea, et al., 2024, for a similar approach), in which participants were presented with brand names embedded within logos. Depending on the condition, the logos either preserved their typical visual format or were modified in font or letter case. Specifically, we compared VWFA activation across three contrasts: (a) brand names presented in a modified font versus their intact visual format, (b) a modified letter case versus the intact visual format, and (c) modified font versus modified case. This design isolates the neural impact of font and case modifications while controlling for the overall logo layout. To rule out the possibility that participants relied on visual format rather than semantic content, we also included mismatched task trials (for details, see Materials and Methods).

Font and letter case are both surface properties, but they differ importantly in their structure. Letter case variations include a highly constrained set of overlearned allographic mappings, whereas font variations span a much larger space of systematic and idiosyncratic shape differences. Our aim is therefore not to equate the two manipulations in perceptual degrees of freedom, but to test whether the reading network treats them differently in the particular case of brand names, where typeface is often part of the item’s learned visual identity. Although both manipulations (letter case and font) affect surface features, previous work suggests that letter case may also serve orthographic roles, signaling lexical category (compare Mary versus mary), as in the “orthographic cue hypothesis” (Peressotti et al., 2003; see also Sulpizio & Job, 2018, for ERP evidence). Supporting this idea, a recent ERP study showed distinct electrophysiological signatures for font-modified versus case-modified brand names (Labusch et al., 2026). While this ERP experiment informs the temporal dynamics of processing, it leaves open whether these effects reach the VWFA. We therefore ask whether this region responds differently to these two types of visual changes, which is a central question to the debate over visual versus abstract coding in visual word recognition.

We framed the experiment as testing a strong version of strict format invariance against accounts that allow residual sensitivity to learned surface forms. If processing in the canonical VWFA ROI is fully normalized with respect to the present manipulations, activation should remain stable across intact and visually modified versions of the same brand name (Cohen et al., 2000, 2002; Dehaene et al., 2005; Grainger et al., 2008).

In contrast, if learned surface regularities continue to influence processing in the VWFA region, modifications in font or case should elicit increased activation relative to intact presentations. This outcome would be compatible with hierarchical, hybrid, or interactive accounts of visual word recognition, in which abstract and surface-form information can coexist, especially for items whose typical visual format is tightly linked to their identity. Such increases in the VWFA activation would be consistent with a prediction error, that is, a mismatch between the incoming perceptual input and learned expectations about the item’s typical visual form, in line with previous behavioral and ERP results (Labusch, Duñabeitia, & Perea, 2024; Labusch et al., 2026). They would also be compatible with episodic or instance-based accounts of word recognition, in which recognition is guided by memory traces shaped by prior exposures (Jamieson et al., 2022; Tenpenny, 1995). However, this pattern would not by itself rule out hierarchical abstractionist accounts that allow residual sensitivity to learned surface form within the ventral occipitotemporal cortex. Rather, it would indicate that, for words with consistent and distinctive visual identities, surface-form information can remain functionally relevant alongside more abstract representations.

As a secondary question, we examined whether font and case modifications evoke similar or dissociable neural responses. If font and case are processed functionally equivalent, they should produce overlapping activation patterns, consistent with models that treat both as irrelevant surface details (Cohen & Dehaene, 2004; Dehaene et al., 2005). However, if they differ in their diagnostic value for brand identity, distinct patterns should emerge, suggesting functional differentiation, with case in particular serving as an orthographic cue that can signal lexical category (Peressotti et al., 2003). Unlike the intact versus modified contrasts, this direct comparison allows for either direction of effect, with stronger activation for font than case, or the reverse.

In sum, brand names provide a stringent test because their canonical visual form is part of their identity. We therefore asked whether disrupting font or letter case, while keeping orthographic identity and semantics constant, modulates responses in the VWFA. Unlike prior work in German nouns and adjectives, where capitalization also cues grammatical class, our manipulations leave lexical category unchanged. Critically, while we expected both font and case modifications to incur a behavioral processing cost (i.e., slower response times), as seen in prior experiments (see Labusch, Duñabeitia, & Perea, 2024), the key question was whether this cost would be uniformly mirrored in the VWFA, or whether the region would selectively respond to one type of modification (font or case). We used a semantic categorization task, which requires direct access to semantic content and reduces reliance on purely visual familiarity, and compared font-modified, case-modified, and intact brand names.

## MATERIALS AND METHODS

### Participants

A total of 32 participants took part in the study. We chose this sample size a priori to match Wimmer et al.’s (2016) VWFA study (*n* = 26 participants) and to be in line with typical samples in event-related reading fMRI experiments. Three participants were excluded due to excessive in-scan motion, defined as more than 2 mm of translation or 2° of rotation in any direction. The final analyzed sample consisted of 29 participants (mean age = 23.66 years, *SD* = 3.99 years, 15 women and 14 men). All participants were right-handed native Spanish speakers with normal or corrected-to-normal vision and reported no reading and/or writing problems and no history of neurological or psychiatric disorders. All of them had normal anatomical MRIs. Participants gave informed consent to participate in the study and received monetary compensation for their participation. The study followed the Declaration of Helsinki and was approved by the Research Ethics Committee of the University of Valencia.

### Materials

**Figure 1.**
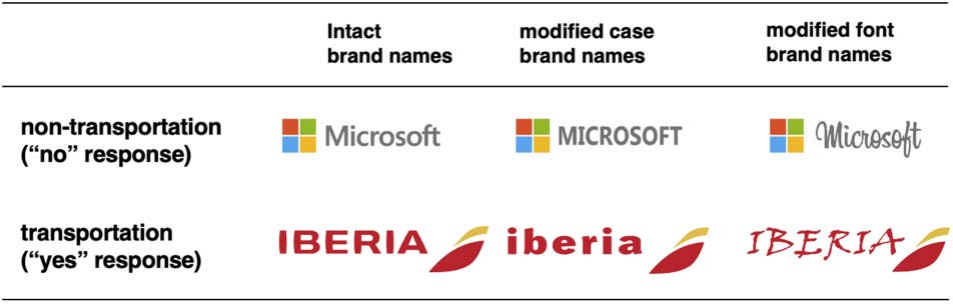
Example stimuli of the semantic categorization experiment, in which participants had to indicate whether the presented brand name was associated with a means of transportation or not.

The target stimuli consisted of 36 brand names presented within their logos. Half of them were brand names associated with a means of transportation (e.g., trains, airlines, buses, car brands), and the other half consisted of brand names from different sectors that were unrelated to transportation (e.g., technology, fashion, social media, food industry, retail). Each brand name was presented within its logo in three possible formats: (a) in its intact, canonical format, (b) with a modified letter case (i.e., uppercase brand names were presented in lowercase letters and lowercase brand names were presented in uppercase letters) but in the original font, and (c) with a modified font that significantly differed from the original font, but maintaining the original letter case configuration (see Figure 1 for examples). All other visual elements were maintained as stable as possible. For the font-modified brand names, we selected one alternative typeface drawn from another real brand logotype in our stimulus set, with the goal of maximizing perceptual dissimilarity from the canonical typeface. These changes typically involved differences such as serif versus sans serif, bold versus non-bold, or thin versus thick strokes. We did not introduce systematic changes in font size, underlining, or italics beyond what was intrinsically part of the canonical logotype, so the intended manipulation was font rather than size or style.

To ensure that participants did not take their decision purely on the basis of the visual information surrounding the brand name, and to ensure reliance on lexical-semantic processing rather than just visual form, we also included a set of 12 mismatched filler brand names, in which the logo did not correspond to the written brand name. In half of these mismatched brand names, a transportation brand name was presented in a non-transportation logo (e.g., the car-brand *Opel* was presented in the logo of the fashion brand *Puma*; “yes” response) and in the other half of the mismatched brand names, a non-transport brand name was presented in a transportation logo (e.g., the fashion brand *VANS* was presented in the logo of the car brand *BMW*; “no” response). Mismatched trials were included only as task-monitoring fillers and were modeled separately as a regressor of no interest in the fMRI analyses (see general linear model [GLM] specifications below), as there were too few trials per subtype to support reliable subtype-specific estimates.

### Procedure

The task was programmed with e-prime software (Psychology Software Tools, Pittsburgh, U.S.). During the fMRI experiment, participants were presented with 144 brand names, each of them presented individually via MRI-compatible goggles (VisuaStim Digital, Resonance Technology) at a resolution of 800 × 600 pixels. Participants were instructed to decide whether the presented brand name was a means of transportation or not by pressing a button of a response box (NordicNeuroLab, Bergen, Norway) with their right or left index finger. Half of the participants had to press the button with their left index finger for the “yes” response and the right index finger for the “no” response, and in the other half of the participants, this order was reversed.

There were three functional runs, each consisting of 12 trials with intact brand names, 12 trials with font-modified brand names, 12 trials with case-modified brand names, and 12 trials with mismatched brand names (48 trials per run). Half of the brand names were transportation brand names (“yes” response), and the other half were non-transportation brand names (“no” response). The order of runs was randomized across participants. Across the three runs, each participant was presented each brand name three times in total, once in each visual format (intact, font-modified, case-modified), with the assignment of format to run counterbalanced, following a Latin square manner, across items and participants.

For each run, stimuli were presented in an event-related design in a randomized order. The first trial of each run was preceded by a 12-second fixation cross due to the scanner’s configuration. Then, each trial started with the presentation of an individual stimulus in the middle of the screen for 1,500 ms. Afterwards, a fixation cross served as an intertrial interval with a mean duration of 3.5 s, varying between trials. It was calculated with the program “fMRIsim” (https://github.com/neurolabusc/fMRI-Simulator) to optimize jittering for estimation of the hemodynamic response function. The trial length varied between 1.62 s and 7.4 s, with an average trial length of 5 s. In total, the experiment took approximately 12 minutes.

After the fMRI task, participants completed a short questionnaire assessing brand familiarity. They were asked to indicate whether any of the 36 experimental brand names were unfamiliar, and to list them if so. Fourteen participants reported recognizing all 36 brands. The remaining 15 participants reported unfamiliarity with one or more brands (*mean* = 1.6 unfamiliar brands).

### Image Acquisition and Processing

Functional neuroimaging data were collected on a 3T General Electric Signa Architect magnetic resonance imaging (MRI) scanner. First, a 3D structural MRI was obtained for each participant using a T1-weighted BRAVO sequence (300 sagittal slices; TR/TE = 7.4/2.9 ms; matrix = 240 × 240; flip angle = 8°; voxel size = 0.5 × 1 × 1 mm; interslice gap = no gap; inversion time = 600 ms). To acquire the functional data, we used a multiband gradient-echo T2*-weighted echo-planar imaging sequence (122 volumes per run, 45 axial slices tilted at approximately 10° from the AC/PC plane; multiband factor = 2; TR/TE = 2,000/23.5 ms; matrix = 64 × 64; flip angle = 80°; voxel size = 3.75 × 3.75 × 3 mm; no gap).

The functional images were preprocessed using the Statistical Parametric Mapping software (SPM12; Wellcome Trust Centre for Neuroimaging, London, UK) and MATLAB (R2022b; MathWorks, Natick, MA) and followed the standard pipeline that included the following steps: reorientation to AC-PC, head motion correction, correction of field inhomogeneity artifacts using the HySCO toolbox (https://www.diffusiontools.com/documentation/hysco.html). Next, slice timing correction was applied to correct the differences in acquisition times across slices, followed by T1 coregistration to the mean functional image, and segmentation. Finally, functional images were normalized to MNI space using the deformation fields from segmentation and smoothed with a 6-mm full-width-at-half-maximum Gaussian kernel. A GLM was defined for each participant using condition regressors convolved with the canonical hemodynamic response function, along with six motion realignment parameters as nuisance regressors. A high-pass filter (128 s) was applied to remove low-frequency components. The analyses focused on experimental trials (font-modified, case-modified, and intact brand names). Mismatched logo-word pairs were modeled as a regressor of no interest. In addition, for each participant, each experimental trial containing brand names they reported as unfamiliar was modeled as a separate regressor and excluded from the main analyses, to ensure that all critical contrasts involved familiar brand names. On average, this procedure led to the exclusion of 2.9% of experimental trials. The following contrasts were performed: (a) font-modified brand names versus brand names presented in their intact visual format, (b) letter case-modified brand names versus intact visual format, and (c) modified font versus modified case brand names.

### ROI Definition

The region of interest (ROI) was defined a priori as a 6-mm radius spherical ROI centered at MNI coordinates (*x* = −45, *y* = −57, *z* = −12). These coordinates were selected following Chen et al. (2019) and Jobard et al. (2003), as they corresponded to the standard canonical peak of the VWFA.

### Statistical Analyses

ROI analyses were performed to examine whether letter case and font manipulation in brand names led to increased neural activity in the VWFA compared to the intact presentations. Marsbar toolbox (https://marsbar.sourceforge.net/) was used to extract condition-wise percent signal change (PSC) relative to baseline for the intact, font-modified, and case-modified conditions. For inferential analyses, corresponding within-subject ROI PSC contrast estimates (modified font vs. intact, modified case vs. intact, and modified font vs. modified case) were entered into one-sample *t* tests against zero in JASP (Version 0.19.1.0; JASP Team, 2026). Statistical significance was set at *p* < 0.05, and *p* values were Holm-adjusted to control for multiple comparisons across the three planned ROI contrasts. We also computed Bayes factors to obtain a complementary measure of the strength of evidence, using Bayesian one-sample *t* tests on the same contrast values in JASP. Bayes factors measure the relative evidence that the data provide for the null versus the alternative hypothesis. Specifically, BF_10_ indicates how much more likely the observed data are assuming the alternative hypothesis compared to the null hypothesis.

As a descriptive control analysis, we examined whether the reported effects persisted when condition-specific mean RTs were entered as covariates in a repeated-measures ANCOVA on ROI activity, with Condition (modified font, modified case, intact) as a within-subject factor. Planned pairwise comparisons between conditions were performed with Holm correction for multiple comparisons.

**Figure 2.**
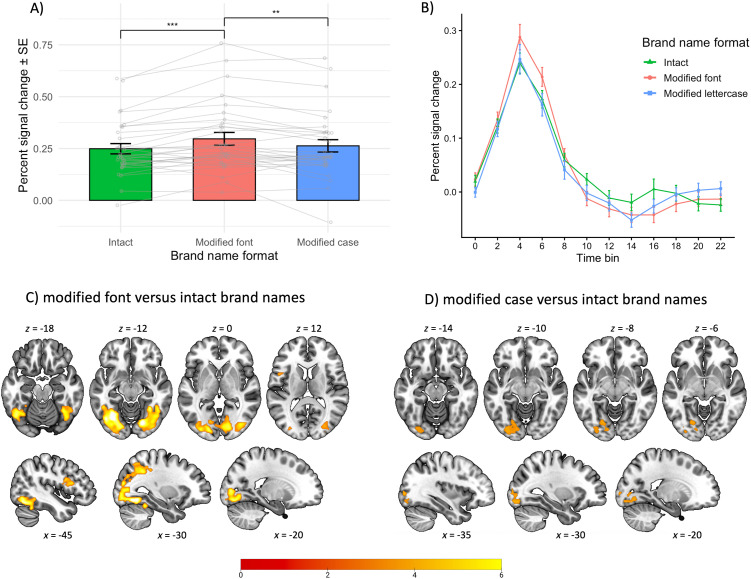
Panel A shows condition-wise mean percent signal change in the visual word form area, with individual participants connected across conditions and error bars showing standard errors. Panel B displays the corresponding time courses of the estimated BOLD response and error bars across twelve 2-s time bins for each experimental condition, based on finite impulse response modeling. Panel C shows the voxel-wise whole-brain analysis for font-modified versus intact brand names. Panel D shows the voxel-wise whole-brain analysis for case-modified versus intact brand names. Color bars represent *t* values.

While our focus was on the activation of the VWFA, we also conducted exploratory whole-brain analyses and condition-specific Finite Impulse Response estimations of the hemodynamic response. These latter analyses are used for illustration only and are not central inferential tests (see Figure 2B), but they provide a fuller picture of whether font and case manipulations differ in amplitude or temporal profile. For the whole-brain analyses, we used second-level voxel-wise whole-brain one-sample *t* tests for each contrast. Statistical thresholds were set at a voxel-wise threshold of *p* < 0.001, with cluster-level correction for multiple comparisons using family-wise error (FWE) at *p* < 0.05. Behavioral analyses were conducted on response times and accuracy values, using paired *t* tests in R (R Core Team, 2025). For each of these measures, pairwise comparisons were conducted between experimental conditions. All *p* values are Holm-corrected for multiple comparisons, with a significance threshold of *α* = .05. Incorrect trials and very short responses (less than 250 ms) were removed from the latency analyses.

## RESULTS

### Behavioral Results

Participants correctly identified transportation brand names in 95.7% of cases and non-transportation brand names in 96.7% of cases. Descriptive statistics are given in Table 1.

**Table 1.** Behavioral results of the semantic categorization task. The table shows the mean response times in milliseconds and accuracy in percent.

	Response Times (in ms)	Accuracy (in %)
Intact brand names	774	97.4
Font modified brand names	813	95.3
Case modified brand names	799	96.0

Correct response latencies were significantly shorter for intact brand names compared to those with font modifications (*t*(28) = 6.26, *p* < 0.001, *d* = .59) or case modifications (*t*(28) = 4.59, *p* < 0.001, *d* = .35); differences between case-modified and font-modified brand names did not reach statistical significance (*t*(28) = 1.97, *p* = 0.059, *d* = .19).

Accuracy in the task was very high (above 95%; see Table 1). The accuracy analysis showed that brand names presented in their intact form were recognized more accurately than those with font modifications (*t*(28) = −3.02, *p* = 0.016, *d* = −.59). The difference between intact and case-modified brand names was not significant after Holm correction (*t*(28) = −2.131, *p* = 0.084, *d* = −.41), and differences between case-modified and font-modified brand names were not significant (*t*(28) = −0.927, *p* = 0.362, *d* = −.19).

### ROI Results

In the VWFA, we found increased activation for the font-modified brand names than for the intact brand names (*t*(28) = 4.33, *p* < 0.001, *d* = .81; BF_10_ = 161.4). In contrast, case-modified brand names did not show a reliable modulation relative to intact brand names (*t*(28) = 1.83, *p* = 0.078, *d* = .34). Bayesian evidence for the modified case versus intact activation revealed only anecdotal evidence (BF_10_ = 0.85), indicating that the data are nearly equally compatible with the null and alternative hypotheses. Additionally, font-modified brand names led to increased activation compared to the case-modified brand names (*t*(28) = 2.62, *p* = 0.028, *d* = .49; BF_10_ = 3.45; see Figure 2A for descriptive statistics). Exploratory supplementary analyses using anterior and posterior VWFA coordinates (Lerma-Usabiaga et al., 2018; Vogel et al., 2012) showed a reliable effect of font in both subregions and a smaller effect of letter case that reached significance in the posterior VWFA, but not in the anterior VWFA; full results are reported in the Supplementary Materials (Supporting Information can be found at https://doi.org/10.1162/NOL.a.268).

### Effect of RT

A repeated-measures ANCOVA including condition-specific RT as covariates showed that the main effect of condition was not significant, *F*(2, 50) = 0.60, *p* = 0.553 after controlling for RT. Importantly, planned pairwise comparisons revealed that the font modified brand names still differed significantly from both intact (*p* < 0.001) and case modified (*p* = 0.012) brand names, whereas case modified and intact brand names did not differ (*p* = 0.095). Thus, the font-related VWFA difference was not eliminated in this control analysis. We prefer to interpret this result cautiously, because RT and BOLD response are themselves consequences of the manipulation rather than fully independent factors.

### Exploratory Whole-Brain fMRI Results

A one-sample *t* test revealed that brand names with a modified font elicited significantly greater activation than intact brand names in several regions: a large cluster (−32, −68, −12, *t* = 7.56, *k* = 5750 voxels; *p* < 0.001, FWE cluster-corrected) extending from the superior parietal lobe to the bilateral occipital lobe, including the left fusiform gyrus and the right lingual gyrus. Two additional significant clusters were observed in the right (42, 14, 16; *t* = 5.17, *k* = 227 voxels; *p* < 0.05; FWE cluster-corrected) and the left (−50, 14, 20; *t* = 4.72, *k* = 355 voxels; *p* < 0.01, FWE cluster-corrected) inferior frontal gyrus (see Figure 2C). The modified letter case versus intact comparison showed a significant cluster in the left middle occipital gyrus (−34, −92, 4; *t* = 4.95, *k* = 428 voxels; *p* < 0.001, FWE cluster-corrected; see Figure 2D), indicating greater occipital activity for logos with modified letter case than for intact logos.

In addition, brand names with a modified font elicited significantly greater activation than brand names with a modified letter case in the bilateral inferior occipital gyrus, specifically in the right (40, −62, −16, *t* = 5.21, *k* = 186 voxels, *p* = 0.03, FWE cluster-corrected) and the left fusiform gyrus (−34, −58, −12, *t* = 4.60, *k* = 180 voxels, *p* = 0.034, FWE cluster-corrected). No significant differences were found for case-modified versus font-modified comparisons.

## DISCUSSION

Brand names provide a critical test case for models of visual word recognition because they challenge the natural tendency of the reading system toward abstraction. Unlike common words, which are encountered in highly variable surface forms and thus may force the cognitive system to normalize the visual input, brand names are typically experienced in a highly consistent visual format. Behavioral and ERP studies have shown that disrupting this canonical format slows recognition and modulates electrophysiological activity even at relatively late stages of processing (Labusch, Duñabeitia, & Perea, 2024; Labusch et al., 2026), suggesting that specific perceptual features may be bound to the lexical representations of brand names. The present fMRI experiment brings this question to the VWFA, examining how font and case modifications affect neural responses to familiar brand names and providing fMRI evidence that these two manipulations can have dissociable neural signatures within the reading network.

Font alterations produced increased activation in the canonical VWFA ROI, whereas case alterations did not show a similarly reliable pattern, despite both manipulations producing slowed response times. Direct comparison confirmed that these two surface features are not equivalent: font modifications led to greater activation than case modifications in ventral occipitotemporal cortex. This pattern is difficult to reconcile with a strong claim that the VWFA is uniformly insensitive to all types of visual format, at least for items whose canonical font is strongly tied to identity (i.e., brand names). Instead, the present data suggest greater sensitivity to font than to letter case, rather than supporting a categorical claim of case invariance.

This conclusion qualifies the dominant view arising from experiments on common words, where VWFA activity has often been described as relatively robust to changes in font and case (see Dehaene et al., 2004; Polk & Farah, 2002). At the same time, more recent research has identified partially separable font-invariant and font-sensitive neural populations in occipitotemporal cortex (Zhou et al., 2019). Thus, these findings are consistent with abstractionist accounts in which word recognition is mediated by case- and font-invariant letter identities (Dehaene et al., 2005; Grainger et al., 2008), while indicating that perceptual information may continue to influence processing within occipitotemporal cortex. We propose that invariance is an adaptive response to input variability. When words are encountered in multiple visual forms, as occurs with common words, the reading system abstracts away surface features that are not systematically predictive. However, the homogeneity of the visual input in brand names may make such abstraction less necessary, allowing specific font information to remain encoded as part of their representation. Importantly, we do not interpret this pattern as exclusive to brand names. Font-sensitive responses have also been reported for regular words (e.g., Zhou et al., 2019) and brand names provide a particularly stringent scenario because their canonical typeface is tightly linked to the brand’s identity.

Instance-based models offer a natural framework for interpreting this flexibility (Goldinger, 1998; Jamieson et al., 2022). In these accounts, recognition is supported by retrieval of prior episodes that include both identity and form. With common words, exposure across diverse formats may promote abstraction through averaging; with brand names, repeated encounters in a stable visual form may reinforce perceptual detail and render it functionally relevant. The VWFA can thus be seen as modulated by both abstract and episodic elements: capable of abstract coding, but also sensitive to perceptual codes when they are relevant for their retention (see Bowers, 2000, for a similar view). A recent high-resolution fMRI study in common words reported that the occipitotemporal cortex contains partially separable font-invariant and font-sensitive neural populations (Zhou et al., 2019). Within a predictive coding account (Price & Devlin, 2011), this flexibility can be interpreted as reflecting stronger top-down predictions for canonical brand formats and increased prediction error when the characteristic font is violated.

The present data do not provide clear evidence that case changes modulate the canonical VWFA ROI under the present task. Given that the Bayes factor for the case-versus-intact contrast was close to 1, this comparison should be treated as inconclusive rather than as evidence for genuine case invariance. While font alterations elicited robust VWFA activation, case alterations did not show a clear effect in the canonical ROI, suggesting that these two surface features may not engage the VWFA in the same way. At the same time, the supplementary subdivision analysis indicates that this conclusion should not be overstated, because the case-versus-intact contrast reached significance in the posterior VWFA. A plausible explanation for this pattern is that normalization of letter case operates via highly overlearned and largely feed-forward mechanisms (see Dehaene et al., 2005). While this process incurs a processing cost, reflected in the slower reaction times (see Table 1), it may be only weakly expressed at the level of the canonical VWFA ROI measured here. This interpretation is consistent with electrophysiological masked-priming experiments with common words, which show case effects only at early perceptual components (N/P150) but not at orthographic or lexical stages (N250, N400; Vergara-Martínez et al., 2015, 2020). At first sight, this seems to contrast with Wimmer et al.’s (2016) letter-case effects in German; however, that dissociation may partly reflect orthographic conventions: in German, capitalization signals grammatical class, so case is not merely a visual attribute. In brand names, by contrast, letter case does not cue grammatical class or lexical category, which may have reduced its functional relevance relative to font in the present task. Within a predictive-coding account (e.g., Price & Devlin, 2011), case modifications may generate a weaker prediction error than font deviations, whereas font deviations, which are more diagnostic of a brand’s canonical form, may behave more like violations of learned visual identity and thus produce a larger VWFA response.

In addition, exploratory whole-brain analyses revealed broader effects of font changes, extending into the bilateral occipitotemporal cortex, parietal regions, and bilateral inferior frontal gyri. These additional activations likely reflect increased perceptual and attentional demands when a brand’s expected format is violated, with parietal regions contributing to a top–down allocation of attention (Corbetta & Shulman, 2002) and inferior frontal regions supporting compensatory phonological or decision processes (Carreiras et al., 2009). In this sense, the effect in the VWFA for font changes appears to be embedded within a broader network response to violations of canonical visual identity.

Taken together, the present findings indicate that font and case are not equivalent in visual word recognition. While there is limited evidence for a VWFA case effect, the present data show that sensitivity to font can remain functionally relevant when a lexical item is encountered in a highly consistent visual format. This pattern is difficult to reconcile with the strongest versions of models of visual word recognition that treat all surface features as equivalent and transient and is compatible with a more flexible account in which perceptual detail is preserved when it is consistently predictive.

The present findings may also be relevant to applied work on typography and brand identity (e.g., see Velasco & Spence, 2019; Velasco et al., 2015). However, because we did not measure consumer evaluation or market behavior, we restrict our interpretation to perceptual and lexical processing: altering a canonical brand typeface changed semantic-categorization performance and neural responses in ventral occipitotemporal cortex. Whether such processing differences translate into brand preference, memorability, or marketplace outcomes remains an open question for future research.

Despite this theoretical and applied impact of the current findings, the present study also comes with limitations. First, the final sample size of 29 participants is typical for event-related fMRI research in reading, but limits sensitivity to smaller effects, particularly for contrasts that were inconclusive in the current data (e.g., the case-modification contrast). Second, our VWFA ROI was defined a priori, using group-level coordinates rather than individualized functional localizers, and may therefore encompass multiple functional subcomponents within the ventral occipitotemporal cortex. Accordingly, this ROI should not be interpreted as mapping onto a single, functionally uniform stage of orthographic processing. To address this limitation, we conducted supplementary ROI analyses using an alternative set of widely used anterior and posterior VWFA coordinates (Lerma-Usabiaga et al., 2018; Vogel et al., 2012). These analyses converged with the main ROI analysis in showing a reliable font effect in both subregions but yielded a less uniform pattern for letter case: the case-versus-intact contrast was not reliable in the anterior VWFA but reached significance in the posterior VWFA (see Supplementary Materials). These supplementary findings should therefore be treated as exploratory and suggest that sensitivity to perceptual manipulations may vary within the VWFA itself. Third, the font manipulation inevitably combined several dimensions of font changes (e.g., serif versus sans serif, stroke thickness, boldness). Given the stimulus constraints, we could not fully balance these visual characteristics across items, so that the observed font effect in this study should be interpreted as a change in typeface, rather than the influence of any single font feature. Future research could systematically isolate the contributions of serif versus sans-serif styles and of stroke-thickness variations in brand-name processing. Fourth, the design did not include a matched non-brand word condition. As a result, the present experiment cannot determine whether the observed font–case dissociation is specific to brand names or reflects a more general property of visually familiar written stimuli.

In conclusion, we found a robust effect of font modification in the canonical VWFA ROI, whereas evidence for case effects was weaker and less consistent across analyses. This pattern constrains those models that make strong claims of complete format invariance, suggesting that perceptual features may remain functionally relevant for some lexical items, particularly those encountered in a highly consistent visual format. Overall, these results indicate that VWFA responses to visually stable brand names are more sensitive to font than to letter case, consistent with the view that perceptual detail can contribute to orthographic processing when it is repeatedly associated with a given lexical item.

## FUNDING INFORMATION

Melanie Labusch, Conselleria de Cultura, Educación y Ciencia, Generalitat Valenciana (https://dx.doi.org/10.13039/501100014849), Award ID: CIAPOS/2024/171. Manuel Perea, Ministerio de Ciencia e Innovación (https://dx.doi.org/10.13039/501100004837), Award ID: PID2023-152078NB-I00. Manuel Perea, Conselleria de Cultura, Educación y Ciencia, Generalitat Valenciana (https://dx.doi.org/10.13039/501100014849), Award ID: CIAICO/2024/198. Victor Costumero, Ministerio de Ciencia e Innovación (https://dx.doi.org/10.13039/501100004837), Award ID: PID2023-152910NB-I0. Victor Costumero, Ministerio de Ciencia e Innovación (https://dx.doi.org/10.13039/501100004837), Award ID: RYC2021-033809-I.

## DATA AND CODE AVAILABILITY STATEMENT

The stimuli, data, and analysis code of the experiment are available at https://osf.io/9a2jv/overview?view_only=5990d8eb20d4451eae8628eb86cf7b14.

## Supplementary Material



## TECHNICAL TERMS

Visual Word Form Area (VWFA): A region in the left ventral occipitotemporal cortex, near the fusiform gyrus, that is involved in the processing of printed words and orthographic representations during reading.
Visual word recognition: The cognitive process of identifying written words and accessing their lexical representations, thereby enabling phonological and semantic processing.
Abstract orthographic representations: Mental representations of written words or letter strings that are relatively invariant to surface visual features (e.g., font, case, and size) and that encode stable orthographic codes.
Orthographic processing: The processing of written word forms, including the encoding of visual features relevant to print, letter identity, letter position, and higher-order orthographic structure.
Lexical representations: Stored word-level representations in the mental lexicon that include orthographic, phonological, semantic, and potentially syntactic properties.
