# Supplementary material for: A Matter of Font: Visual Word Form Area Responses to Brand Names Are More Sensitive to Font Than to Letter Case Changes
Source: Neurobiol Lang (Camb). 2026 Jul 1;7:NOL.a.268. doi: 10.1162/NOL.a.268 (PMC13379300; doi:10.1162/NOL.a.268)
Supplement: Supplementary file 1 [file nol-07-268-s001.pdf]

Supplementary Materials.

To further examine possible functional heterogeneity within the VWFA, we conducted exploratory ROI analyses using anterior and posterior VWFA coordinates derived from Vogel et al. (2012) and Lerma-Usabiaga et al. (2018). Mean contrast values were extracted from the anterior VWFA (MNI: -45, -51, -12) and posterior VWFA (MNI: -45, -72, -10), and each contrast was tested against zero across participants.

Table S1. One-sample t-tests on the anterior and posterior parts of the VWFA, according to coordinates by Vogel et al. (2012) and Lerma-Usabiaga et al. (2018) for each experimental contrast.

	Anterior VWFA (MNI: -45, -51, -12)			Posterior VWFA (MNI: -45, -72, -10)		
contrast	*t(28)*	*p*	*d*	*t(28)*	*p*	*d*
Font-modified vs. intact brand names	4.139	<.001	.77	5.513	<.001	1.02
Case-modified vs. intact brand names	1.919	.065	.36	2.533	.017	.47
Font-modified vs. case-modified brand names	2.536	.017	.47	2.885	.007	.54

As shown in Table S1, the contrast between font-modified and intact brand names was reliable in both anterior and posterior VWFA. The contrast between case-modified and intact brand names was smaller overall and reached significance only in the posterior VWFA. Finally, the contrast between font-modified and case-modified brand names was significant in both subregions. These exploratory analyses therefore suggest that sensitivity to font-based perceptual variation was present across both anterior

and posterior portions of the VWFA, whereas sensitivity to case-based variation was more limited and appeared to be more evident in posterior VWFA.
